# Remarks on the History and Use of Tobacco

**Published:** 1810-12

**Authors:** 


### Medical and Physical Journal.

VOL. XXIV.] DECEMBER, 1810. [NO. 142.

Printed for R. PHILLIPS, by E. Hemsted, Great New Street, Fetter Lane, London.

### *To the Editors of the Medical and Physical Journal.*

### *Remarks on the History and Use of Tobacco.*

GENTLEMEN,

THE powers and the properties of the vegetable narcotics, either to destroy or to save, have been enough distinguished, to make those who wish for the improvement of medical science, lament that our knowledge of them is still so indeterminate, that it may be said to approximate to ignorance. Some of the properties of some of these substances, may have been tolerably explained; but of others, so little is ascertained, that they still remain, as to their influence upon the animal functions, in a state of great obscurity. On Opium, with which our acquaintance is most perfect, much has been written; with it many experiments have been made; and numerous observations respecting it have been recorded. But even of this substance, which is procured from the most familiar of this tribe of soporific plants, our knowledge is imperfect and unsatisfactory. Of the Digitalis our ignorance was profound, before the publication of Withering's pamphlet. Of the Solanums, Datura, Hyoscyamus, Conium, Cynoglossum, the Agarics, in some of their species, what do we know? We are certain, however, that many individuals of the LURIDÆ are absolute and direct poisons, but in a quantity short of the poisonous dose, they may be powerful remedies. If this is not a new fact, it is one of great importance, and deserves the most serious attention. All these plants, or parts and preparations of them, have, certainly, at various times, been employed as remedies; yet one misfortune has accompanied this employment. Either from some overweening partiality, or from some happy effect, accidentally perhaps produced, or connected with some unascertained cause, their patrons have given them a character above their deservings: and having always been found to fail in the performance of wonders, their reputation has fallen into the other extreme; and that has been rejected as useless, whose powers were not found to be miraculous. But let us rather admit, as a more fair and rational conclusion, that these substances, when they have failed or been injurious, have been applied to unappropriate diseases; that their dose has been either too large or too small; or that there has been some circumstance in the case at their time of exhibition, unpropitious to their use. To ascertain which are the proper cases, what is the proper dose, or which the temporary symptoms that may forbid their use; and how those symptoms are to be removed before they are admissible, becomes the serious duty of the medical practitioner. Neither has it been determined, possibly not much considered, whether, if compounded with each other, in various proportions as well as kinds, a medicine might not be produced, having properties different in degree or quality; and, perhaps, more under controul than either of the separate substances.

These reflections have arisen from reading some observations upon Tobacco in your Journal for November, full of point, and where, possibly, "more is meant than meets the ear."

But, if I acknowledge the writer's talent, I am not compelled to submit to his conclusion, unless his argument convinces.—He is an enemy to TOBACCO, from having been disappointed, perhaps, in its expected effects, or from some latent prejudice; yet am I not without hope to shew, even to a descendant of *Joshua Sylvester*, that this plant possesses properties that may be employed with a fair prospect of being successful in the cure of diseases.

It has been the fate of the NICOTIANA TABACUM to have credulous and hyperbolical friends; enemies prejudiced, malignant, and unjust. With the one party it was the great *Panacea*,* the curer of every evil, the soother of every care.—With the other, it was a debaser of the human mind, enervated the body, and was fit only to be used by diabolical

* A book was written by Dr. Giles Everard, (a Dutch Physician, born at Berg-op-Zoom,) and published in 1583, with the title *De herba Panacea, quam alii Tabacum, alii Petum aut Nicotianum vocant, brevis commentariolus, quo admirandæ ac prorsus divinæ hujus Peruanæ stirpis facultates et usus explicantur.* Antverpiæ, 16mo. This passed through more than one edition, as will be noticed hereafter; and was formed into an English duodecimo, with the title of *Panacea*, &c. 1659. This last, when it has the portrait, in this portrait-mania age, sells for 15s.

spirits spirits in Pandæmonium.* Both its friends and enemies were found in every rank of society, from the King to the peasant. Poets, priests, physicians, and moralists, were, by turns, its panegyrists and its defamers.

When the exalted genius of Columbus directed him to a new world, believed, by men of baser intellect, then to have no existence but in his own imagination; the wonders of the vegetable kingdom, both in external form and in peculiar properties, were not the least curious and valuable of his discoveries. The TOBACCO PLANT † grew in Cuba, and must

---

* That wise monarch, James the Ist, who, *gude mon*, was hardly quarrelsome on any subject but *Tobacco*, to which he had a mortal hatred, used to say, in his hours of relaxation, that this plant was the lively image and pattern of hell; (vid. a collection of witty Apothegms, &c. 12mo, 1671) for that it had, by allusion, in it all the parts and vices of the world, whereby hell may be gained: to wit, 1st, *it was a smoke*; so are the vanities of this world. 2dly, *it delights those who take it*; so do the pleasures of the world make men loath to leave them. 3dly, *it maketh men drunken and light in the head*; so doth the vanity of the world, men are drunken therewith. 4thly, *he that taketh Tobacco saith he cannot leave it, it bewitcheth him*; even so the pleasures of the world make men loath to leave them, they are so enchanted for the most part with them. And farther, beside all this: 5thly, *it is like hell in the very substance of it, for it is a stinking loathsome thing*; and so is hell." This quibbling sophist, concludes with, "Were I to invite the devil to dinner, he should have three dishes; 1st, a pig; 2d, a pole of ling and mustard; 3d, a *pipe of Tobacco* for digesture." Compared with the royal authour's "COUNTER-BLAST" this is tender mercy.

† I am aware an opinion has prevailed, that *Tobacco* grew in Asia, and was even smoked, long before the discovery of America. *Professor Beckmann*, of Gottingen, whose laborious researches into every species of literature, but especially those connected with physical science, and the invention of arts, are so well known and appreciated, says, (*Introduction to Technology*) the celebrated naturalist and traveller, Pallas, informed him, "that in Asia, and especially in China, the use of Tobacco for smoking was more antient than the discovery of the new world. Among the Chinese, and among the Mongul tribes, who had the most intercourse with them, the custom of smoking is so universal, and has become so indispensible a luxury; the Tobacco-purse affixed to their belt, so necessary an article of dress; the form of the pipes, from which the Dutch seem to have taken the model of theirs, so original; and, lastly, the preparation of the yellow leaves, which are merely rubbed to pieces and then put into the pipe, so peculiar; that we cannot possibly derive all this from America by way of Europe; especially as India, where the habit of smoking Tobacco is not so general, intervenes between Persia and China. May we not expect to find traces of this custom, in the first account of the voyages of the Portuguese and Dutch to China?" That celebrated traveller and philosopher ULLOA, observes (Voyage to South America, Vol. I. 139) "that have been early known to Columbus and his companions. In 1492, possibly, they were acquainted with it. Four years after this *Romanus Pane*, a Spanish monk, whom Columbus, on his second departure from America, had left in that country, published the first account of *Tobacco*,* with which he became acquainted in St. Domingo. But until there is better evidence than at present appears, it must be considered, that this account of *Romanus Pane*, and his publication on Tobacco, deserves little credit.

*Hernandez de Toledo* sent this plant into Spain and Portugal, in 1559, when *Jean Nicot*,† was ambassador at the court of Lisbon, from Francis the IId. and he transmitted, or carried, either the seed or the plant, to Catherine de Medicis. It was then considered, as one of the wonders of the new world; a proper present for a Queen; and was believed to possess extraordinary virtues. This is, probably, the first authentic record of the introduction of this potent plant into Europe; a plant which was afterward to be-

---

it is not probable the Europeans learned the use of Tobacco from America; for as it is very ancient in the Eastern countries, it is natural to suppose, that the knowledge of it came to Europe from those regions, by the means of the intercourse carried on with them by the commercial states of the Mediterranean sea. No where, not even in those parts of America where the Tobacco plant grows wild, is the use of it, and that only for smoking, either general or very frequent." After all, possibly, the Tobacco of Asia, the smoking of which is so antient a custom, is not the *Nicotiana Tabacum*, but the *N. fruticosa*, which nearly resembles it, and grows in China and Cochinchina, is cultivated every where in those countries, has its proper vernacular names, and is regarded as indigenous. In 1699, according to the Hortus Kewensis, N. fruticosa was cultivated here by the Duchess of Beaufort. If the practice of smoking Tobacco came from the East into Europe, why is there no account of it before the discovery of America?

* I acknowledge my ignorance of *Romanus Pane*, the Spanish monk, and of his account of Tobacco; and am indebted to *Schlozeres' Briefwechsel*, for the anecdote. Perhaps some of your learned correspondents may hunt this first writer on the Tobacco plant from his obscurity. If they are successful, it will show how early after the discovery of America, we became acquainted with this narcotic.

† *Nicot*, whose name is now only preserved from oblivion by having been given to an American weed, was a notary in the town of Nismes, in Languedoc; but having the good fortune to be introduced at Court, he became a favourite with Henry II. and Francis II. He was made a master of Requests, and was ambassador to Portugal, in 1559, 1560, and 1561. He died at Paris in May, 1600. His having first brought Tobacco into France, has occasioned his name to be inserted by Eloy, in the "*Dictionnaire Historique de la Medecine.*"

become come the theme of poets, and the admiration of physicians: to be condemned by popes, and execrated by moralists.

The reputation of *Tobacco*, from this period, gradually spread throughout the European states. In 1565, Conrad Gesner,* the Pliny of Germany, became acquainted with it; and it was found in many gardens. In 1570, according to Lobelius (Hort. Kewensis) it was cultivated in England: though Sir Richard Baker (Chronicle, p. 400. Edit. 1696,) asserts, it was brought hither from the West Indies by Ralph Lane, in the 28th year of Elizabeth, 1586.† On this subject, Lobelius is better authority than this credulous chronicler. Baker's account, probably, applies to the smoking of *Tobacco*, and not to its first introduction. And this is still rendered more likely, by the English having, in 1585, first seen tobacco-pipes made of clay, ‡ among the native Indians of Virginia; which was about that time discovered by Richard Greenfield. It appears that the English, soon after this, perhaps in 1586, manufactured the first clay tobacco-pipes in Europe. The Dutch, notwithstanding their dull preeminence in the art of using this narcotic, still smoked out of conical tubes, composed of palm leaves plaited together.

It now quickly made its way over the European continent. In 1575, a figure of the plant, given by André Thevet,§ in his *Cosmographia*, (and which, perhaps, was the first) made it more known, at least to those who entered into the botany of that time. In 1610, smoking tobacco was known and practised at Constantinople. But it seems, there, to have

---

* *Epistolæ Medicinales*, fol. 79, Nov. 5, 1565. He had then only learnt from Thevet, that it was used in America for smoking.

† Another account says, that Sir Francis Drake made tobacco known to the English in 1586. *Panacea*, p. 3.

‡ *Clusius* says, that the English returning from Virginia brought from thence tobacco-pipes made of clay, and since that time "the use of drinking (smoking) tobacco hath so much prevailed all England over, especially among the courtiers, that they have caused many such pipes to be made to drink tobacco with." *Panacea*. *Clusius* had been twice in England, was intimate with Sydney and Drake; and from the latter he procured many materials for his *Exotica*, printed at Antwerp in 1601. It cannot be doubted therefore, when he speaks of the prevalence of smoking in England, that he gives it on his personal knowledge.

§ Thevet (who was in that expedition, which, anno 1555, Nicolaus Durandus Villagagnonus made to Brazil) in his book called Antartick France, boasts, that he was the finder, and the first man that brought the seed of Tobacco into old France. *Panacea*, p. 6.

been been considered as a new-fangled and foreign luxury; and something disreputable was attached to this mode of using it.* The progress of the cultivation and use of this plant throughout the German states, and in the Swiss cantons, is minutely traced in the work of Prof. Beckmann, before cited. In 1620, some Englishmen instructed the inhabitants of Zittau to smoke; and in the same year Konigsmann, a merchant, brought the first Tobacco plant from England to Strasburg. Two Jews attempted its cultivation in the Margraviate of Brandenburg, in 1676. In 1677 great quantities were grown in the Palatinate, and in Hessia. Vicarius,* an Austrian Physician, is said, in 1689, to have improved the common tobacco-pipe, by adding to it a tube which contained sponge, through which the smoke percolated; and was made grateful by an abstraction of a portion of its calorique, or rendered odoriferous by being impregnated with some perfume in its passage.—Smoking was a practice so novel in the canton of Appenzel, in 1653, that children ran after those who used a pipe in the streets. In short, Tobacco excited, wherever it came, the feelings, the passions, and the interests of mankind, in a most extraordinary degree. It gave alarm to both church and state. Religion and morality were thought to be endangered by its use; and the welfare, even the existence of society, was threatened by its cultivation.†

---

* It is related by *Beckmann*, that a Turk, who had been found smoking, was conducted through the streets of Constantinople, with a pipe transfixed through his nose. The Turks were late before they learned to cultivate the plant themselves; and continued a long time to purchase a refuse Tobacco (Mundungus) of the English. Now a very choice and expensive kind (Ellsham) is imported into this country from Turkey.

* Was this *Jean Jacques Vicarius*, a doctor in philosophy and medicine in the University of Fribourg in the Brisgau, and a Member of the Imperial Academy, (*Academiæ Naturæ Curiosorum*) in 1697, under the academical name, Anaximander, who was also a man of great ingenuity, and the author of several works on medical subjects!

† The extent to which the passion for Tobacco went, will afford a reasonable ground for the apprehensions that pressed upon the guardians of religion, morals, and the general welfare of nations. One of its early apologists says, "Seamen will be supplied with it for their long voyages; soldiers cannot want it when they keep guards all night, or are upon other hard duties in cold and tempestuous weather; farmers, ploughmen, porters, and almost all labouring men plead for it, saying they find great refreshment by it, and would as soon part with their necessary food as with Tobacco. The nobility and gentry, who find no fault with it, but that it is too common amongst the vulgar, do ordinarily make it the complement of their entertainments, and oftimes all their entertainment beside is a complement. Scholars use it much, and many grave and great

The effects produced by the *Nicotiana Tabacum* upon the intellectual principle, whether real or imaginary, are of a

---

great men take Tobacco to make them more serviceable in their callings. Tobacco is grown to be not only the physick, but even the meat and drink of many men, women, and children. In a word, it hath prevailed so far, that there is no living without it; that notwithstanding the vast plantations of it in the West-Indies, *all our corn fields would soon be turned into Tobacco-gardens, were not men restrained from it by the civil magistrate.*" This was said of it in 1659. From the beginning of that century the Tobacco-mania was so violent, that it became necessary to restrain it by severe laws.

In 1604,* James the 1st. endeavoured, by means of heavy imposts, to abolish its use in this country. In 1619, this same monarch commanded that no planter in Virginia should cultivate more than 100lbs.†. Pope Urban the 8th published a decree of excommunication, in 1624, against all who took snuff in the church. Ten years after this, smoking was forbidden in Russia, under the pain of having the nose cut off. In 1653, the council of the canton of Appenzell cited smokers before them, punished them, and ordered innkeepers to inform against such as were found smoking in their houses. The police regulation of Bern, made in 1661, was divided according to the Ten Commandments. In it the prohibition to smoking stands immediately beneath the command against adultery. This prohibition was renewed in 1675; and the tribunal instituted to put it into execution (*Chambre au Tabac*) continued to the middle of the 18th century. Pope Innocent the 12th, in 1690, excommunicated all those who were found taking Snuff or Tobacco in the Church of St. Peter at Rome. Even so late as 1719, the Senate of Strasburgh prohibited the cultivation of Tobacco, from an apprehension that it would diminish the growth of corn. Amurath the 4th published an edict, which made smoking Tobacco a capital offence. This was founded on an opinion that it rendered the people infertile.

Whether these laws were all justly founded, or whether they arose out of prejudice, passion, or false opinion, they still shew the estimation in which Tobacco was held.

* The record of this impost, preserved in the 16th Vol. of the *Fœdera*, is valuable not only as a fact, but as an historical document, characteristic of the opinions that prevailed at the commencement of the 17th century. Its title is, *Commissio pro Tobacco*, wherein King James sets forth, "That, whereas Tobacco being a drug of late years found out, and brought from foreign parts in small quantities, was taken and used by the better sort, both then and now only as physick, to preserve health; but it is now, at this day, through evil custom and the toleration thereof, excessively taken by a number of riotous and disorderly persons of mean and base condition, who do spend most of their time in that idle vanity, to the evil example and corrupting of others, and also do consume the wages which many of them got by their labour, not caring at what price they buy that drug. By which immoderate taking of Tobacco the health of a great number of our people is impaired, and their bodies weakened and made unfit for labour. Besides, that also a great part of the treasure of our land is spent and exhausted in this only drug, so licentiously abused by the meaner sort. All which enormous inconveniences we do well perceive to proceed principally from the great quantity of Tobacco daily brought into this our realm, which excess might, in great part, be restrained by some imposition to be laid upon character to interest curiosity, and excite investigation. To this part of the inquiry the elucidation will be afforded, principally, by the enthusiasm of the poets. Those of the *genus irritabile Vatum* who had felt its soothing influence, or had drank with delight of the wild reverie which its operation frequently produced, celebrated, in grateful strains, its potent virtues. Those who had rejected the insidious poison, execrated both the plant and its friends, if not with the courtly elegance of Horace, or the fire of Juvenal, certainly with a redundant portion of the virulence of Persius. Though fiction be the privilege of poetry, yet it has not unfrequently occurred that the poet has painted with a faithful pencil the manners and the customs of mankind; and the historian has transferred to his pages useful materials, collected with proper discrimination, from an art, the essence of which has always been considered as allowing a deviation from the plain path of truth.

Very early, before 1589, the cardinal *Santa Croce*, returning from his nunciature in Spain and Portugal to Italy, carried thither with him Tobacco. This circumstance is commemorated by *Castor Duranti*, in latin verses which lavish

---

upon it. Wherefore we command you our Treasurer of England, to order all customers, comptrollers, &c. of our ports, that from the 26th day of October next, they shall demand and take for our use, of all merchants, as well English as strangers, &c. the sum of six shillings and eight-pence in every pound weight thereof, over and above the custom of twopence upon the pound weight usually paid before."

† This proclamation for restraining the planting of Tobacco in Virginia, was followed by another in 1620, inforcing the former. The preamble to this latter places in a strong light the mortal hatred James had to this herb.—" Whereas we, out of the dislike we had of the use of Tobacco, tending to a general and new corruption both of men's bodies and manners; and yet, nevertheless, holding it, of the two, more tolerable that the same should be imported, amongst many other vanities and superfluities which come from beyond seas, than to be permitted to be planted here within this realm, thereby to abuse and misimploy the soil of this faithful kingdom; and whereas we have taken into our royal consideration, as well the great waste and consumption of the wealth of our kingdoms, *as the endangering and impairing the health of our subjects*, by the immoderate liberty and abuse of Tobacco, *being a weed of no necessary use*, and but of late years brought into our dominions. We therefore strictly charge and command that our proclamation, of December last, restraining the planting of Tobacco, be observed." In 1624, James strictly prohibited, by proclamation, the planting of Tobacco in England or Ireland. In 1627, Charles I. renewed this prohibition. In 1660, the first legal act of parliament was passed against planting Tobacco in England and Ireland. Opinions were now so far changed, however, that the corruption of manners, and the injury to health were no longer the ostensible motive, but the loss the colonies would sustain by it being grown at home—it was allowed to be grown in the physic gardens of the two universities, and in private gardens for surgery. After this, we no longer hear, in this country at least, of apprehensions for the health and morals of the people with regard to the use of Tobacco. All laws, either restrictive or promotive, have henceforward, a commercial object.

the the most egregious praises on this plant: not only is it asserted to cure every disease, but the holy Cardinal's exploit is compared with that of his progenitor, who brought home the wood of the true cross.

"Hanc Sanctacrucius Prosper quum Nuncius esset
Sedis Apostolicæ Lusitanas missus in oras
Huc adportavit Romanæ ad commoda gentis,
Ut proavi Sanctæ lignum Crucis ante tulere
Omnis Christiadum quo nunc respublica gaudet,
Et Sanctæ Crucis illustris Domus ipsa volatur
Corporis atque animæ nostræ studiosa salutis."*

It is probable, especially at this time and in this place, and when thus introduced by a parallel between it and the wood of the sacred cross, that many superstitious and miraculous properties would be given to it. Possibly some of your correspondents of deeper research than I can boast, may discover that Tobacco was then employed in amulets and mystical sigils, consecrated with "muttered spells," to ward off contagion, perhaps to repel witchcraft, and the operations of evil spirits.

Animated as the eulogium of *Duranti* was, it sinks into nothing before the strains of *Raphaelle Thorius*, who, in a Latin poem (*Hymnus Tabaci*†) celebrates the properties of the *Nicotiana* in Virgilian numbers.

"Planta beata! decus terrarem, munus Olympi!
Non tantum agricolis duro lassata labore
Membra levas, minuis victus absentis amorem,
Fundis et absque cibo sparsas in corpora vires.

---

* ———————— Herb of immortal fame!
Which hither first with Santa Croce came;
When he, his time of Nunciature expired,
Back from the court of Portugal retired;
Even as his predecessors great and good
Brought home the cross, whose consecrated wood
All Christendom now with its presence blesses:
And still th' illustrious family possesses
The name of Santa Croce, rightly given,
Since they in all respects resembling heaven,
Procure as much as mortal can do,
The wellfare of our souls and bodies too.

† The intrinsic excellence as well as the scarceness of the *Hymnus Tabaci*, will be an apology for my extracting largely from it. The author, Raphaelle Thorius, who is now but little known, was a Frenchman, a doctor of the University of Oxford, and a physician in London, in the reign of James the 1st. where he died in 1629. The *Hymnus Tabaci* has passed through many editions: It was printed at Leyden, 4to. 1622, probably the princeps, and again, same place, 4to. 1623; and 1628. 12mo. Utrecht, 1644; and London, 12mo. 1651. Two letters "*de causa morbi et mortis de Isaaci Casauboni*," were likewise published by Thorius. With the classical elegance of style which Thorius cultivated *con amore*

But he assigns to it a much higher office than restoring the enervated body, or refreshing the wearied peasant's limbs.

> " Sed radium specimenque Dei sapientibus ipsis
> Ingenium illustras, si quando aut multa tenebras
> Colligit ingluvies cerebro, aut nolimine longo
> Intellectus hiat, rerum neque concipit umbras,
> Conceptasve tenet, vel cœca oblivia regnant;
> Ut semel irrepsit blando lux indita fumo,
> Aufugiunt nubes atræ, curæque, tenaces.
> Vis micat inventrix, dempto velut obice veli
> Tota oculis animi patet ampli machina mundi,
> Æternæ species naturæ ex ordine nexæ
> Succedunt, redeuntque; suis simulacra figures."*

This general illumination of the mental principle, this intellectual sharpening to cut into the deep recesses of nature, until the *species æterna*, the secret stamina of the world st and exposed, is followed by a particularization of the operation of this herb on the orator and the disputant.

> " O quoties visus magna spectante corona
> Orator populi cupidas diuturnus ad aures
> Contremuisse metu, docti sermonis acervos
> Confudisse locis, lingua et silvisse rigenti

---

*amore*, he joined a devotion to wine and tobacco. He was the greatest bon vivant of his time, a hero of the bottle whom no man dared to engage:

> ———————" A doctor of tremendous paunch,
> Awful and deep."

M. Peirese, one of the finest geniusses of the 17th century, being at a dinner in London, with some of the literati of that age, punished Thorius severely by compelling him to drink a large glass of water. Gassendus gives, very pleasantly, the history of this transaction. " Contigit ut in quodam virorum doctorum convivio, Doctor Thorius ipsi Peireskio ingenti Scypho præbiberit: Ac ille quidem se excusare, ob vastitatem pateræ; ob merum insolitum; ob imbecillem stomachum; ob compotandi infrequentiam: verum cum nihil admitteretur, petiit, ut saltem sibi liceret, postquam Thorio fecissit satis, suo arbitrio præbibere. Annuerunt omnes, ac tum assumptis, quasi adigente necessitate animis, fœcundum hausit calicem, eodemque mox aquâ oppleto, Thorio intentans præbibit, totumque rursus (tanquam injectum temperaturus merum) absorpsit. Ille quasi fulmine ictus, delapsusve è nubibus, vix tandem ad se rediit, et quia ex condicto agebatur, neque resilire fas erat, tam longa suspiria è pectore duxit, toties admovit, removitque ora, tot interea carmina ex omnibus Græcis, Latinisque poetis profudit, ut diem penè contriverit instillandæ aquæ in insuetum guttur." *Vita Peireskii. Lib.* 2.

* The Poet's fiction is almost realized by one of the most learned men of this country, (or of any other) being also its greatest smoker. We owe, *Raphaelle Thorius* would have said, the *sesquipedalia verba*, the sounding periods, and the lacerating keenness of Tracts by Warburton and a Warburtonian, to the influence of the *Nicotiana Tabacum.*

Quum

Quum memor ex tantis opibus sopita facultas
Nil daret in vocem, sed res et verba negaret,
Si modo vel micam generosa è stirpe vorassit
Fumanti tubulo, accenso seu lumine, sensim
Res reperisse suas, prendisse fugacia verba
Thesaurosque ; animi populo exposuisse stupenti.

This is a lively picture of the confused and doubting orator, and of the sudden renovation of his faculties by this potent herb. With equal address is the disputant excited to keener contest by its influence; and when defeated, rising from its fumes, like Antæus when he touched the earth.*

The dominion of tobacco over the mind was not always manifested by that fictitious elaboration of intellect, on which this classic poet delighted to dwell. It was more frequently used to disturb the imagination, to produce that wild and savage enthusiasm, which among barbarous tribes is considered as a visible indication of a direct communion with the Deity.* The extacies thus produced were often extremely serious † in their consequences.

---

* "Si duo præterea Stagiræ in pulvere docto
Laudis amore novo, verive cupidine pulsi,
Pugnam agitant, verbis et degladiantur acutis:
Omne animi intendunt robur, nervosque fatigant
Nexibus impediant cautum ut subtilibus hostem,
Aut validum incusso rationem pondere sternant.
Stat circum pubes, pulchri & certaminis æquus
Arbiter ingenii æquales utriumque lacertos
Mirantur, crebris vires applausibus augent.
Tandem obtusa acie, ut fessis deferbuit ardor,
Lentior et validos insultus lucta remisit,
Sorbeat alterutur, sacrum refletque vaporem
Ilicet ut tactâ Antæus tellure, resurgit
Firmior, exhaustumque recens grassatur in hostem;
Jejunus cadit, at sequitur victoria potum.
Sin utrique placet nova de suffimine tela
Arripere, & calidam verbis producere pugnam:
Tum videas paribus momentis prœlia sæva
Eventu dubio in multam crudescere noctem ;
Nec prius argutis discedere ab ictibus ambos,
Indefinitam abscindat quam tædia litem
Judicis incerti, palmæ cui prœmia donet."

* The aborigines of America, according to Harriot's description of them, cast powdered tobacco into their sacrificial fires, believing it grateful to their gods. The priests of some tribes swallowed the smoke of this plant to excite the spirit of divination ; and this they did to a degree that threw them into a stupor of many hours' continuance. When recovered from this paroxysm of drunkenness, they asserted they had

In its operation on the intellects of the inhabitants of Europe, Tobacco was not observed to produce any of these effects. Sottishness, indolence, and torpor, when it was employed to excess, were the common result. By an extravagant fondness for it, men became ruined in their fortunes,* bankrupt in reputation, imbecile in mind, and emasculate in body.†

That this was the opinion held most earnestly by some persons, both history and their existing works afford abundant proof.

Among those who have in this country drawn the pen against *Tobacco*, James the First claims the place of honor, both from his rank, and the energy of his Philippic. The "COUNTER-BLAST TO TOBACCO," (the quaint Title to

---

had conferred with the devil, who had shewn them the course of future events. Their physicians also get drunk with this smoke, and relate, that while under this intoxication, they are admitted to the counsel of their gods ; who reveal to them the event of diseases.

† Monardes says, that the servants and the Moors (negroes perhaps) who came into the West-Indies, took such delight in smoking tobacco, that they appeared to lie as dead from its use, for several hours ; and that sometimes they grew so mad with it, as to kill their masters. It is a curious fact in the history of man, that producing a maniacal disturbance in the brain and nervous system by fumigation, is a practice of great antiquity. The Scythians when they desired to be intoxicated, and yet, in compliance with some custom or law, abstained from wine, cast bundles of herbs into the fire and inhaled the smoke, which produced the effect, (Alexander ab Alexandro, and Max. Tyrius.) The Thracians produced the same effects by the smoke of the seed of some plants. (Pompon. Mela and Solinus.) The Babylonians, according to Herodotus, employed similar means to produce intoxication.

* King James asserts, (Counter-Blast) that some persons spent 4 or 500l. a year in tobacco ; an enormous sum for that time.

† These are the heavy charges brought against this herb by its enemies, whether verse-men or prose-men. They are not, however, warranted by observation, in the present day. That sedate, sober, and capable men smoke tobacco, almost incessantly, must be known to many persons ; neither do their mental or physical powers suffer by it. The drunkard smokes, but his pipe is a mere appendage to his pot. Observation leads to a conclusion that there are more sober than drunken smokers. Smoking does not appear to be quite so innocuous in America ; for there, says a modern traveller, (Janson's Stranger in America, 4to. 1807) children smoke *segars* to so immoderate a degree, that death has been the consequence. This, he adds, is fully elucidated by the following paragraph, copied from a late newspaper, printed at Salem, in Massachusetts :

" *Died*

the royal Penman's production) is both argumentative and declamatory. Four propositions, or reasons for indulging in the use of Tobacco, are stated as the usual grounds of its defence; and argued against fluently enough, according to the scholastic method of that day. Two of the propositions are stated as theoretical, and two are experimental. The theoretical are, 1st, "It being a sure aphorism in Physick, that the brains of all men being naturally cold and wet, all dry and hot things should be good for them; and therefore this stinking fumigation must be useful." The 2d is, that "this filthy smoke, as well through the heat and strength thereof, as by a natural force and quality, is fit to purge both the head and stomach of rheums and distillations, as is proved by spitting and voiding of phlegm." The experimental grounds of the defence of Tobacco are, 1st, "That people would not have taken so general a good liking to it, if they had not by experience found it sovereign and good for them." 2d, "That divers and very many men do find themselves cured of divers diseases, as on the other part no man ever received harm thereby." With the first propositions, being purely and ridiculously hypothetical, there was not much difficulty: with the second, as being founded on the experience of mankind, more ingenuity must be employed. The argument is managed with some dexterity, and turns on taking *non causam pro causa.* We have to lament, that this still happens in deducing conclusions from

---

"*Died in Salem, Master James Verry, aged twelve, a promising youth, whose early death is supposed to have been brought on by excessive smoking of segars.*"

That this pernicious custom was habitual in an infant, not four years of age, Mr. Janson says, he was himself a witness. This little boy is the son of Thomas Taylor, a segar-maker, in Alexandria, near Washington. While conversing with the father, our author observed the son smoking a large segar, made of the strongest tobacco. On expressing his astonishment, the infatuated parent replied, that the child had contracted the habit above a year before, and that he smoked three, four, or more daily: in fact, the child was seldom without a lighted segar in his mouth, and yet he was fat and healthy. (p. 297.) In these two specific instances the evidence is so poised as to be quite indecisive. A boy twelve years old was killed by smoking segars; a child of four years old grows fat and is healthy, under the same practice. Professor Waterhouse, in a public Lecture on the Preservation of Health, (Nov. 1809) at Cambridge, in New England, speaks of tobacco as a treacherous enemy that is secretly doing much mischief; but hesitates to attack it on account of the popular opinion being so greatly in its favour.

premises premises not well understood. After declaiming against Tobacco on its uselessness as a medicine generally, its injury to the frame when in health, and on the profusion of expense its use incurs, the royal author thus concludes the *Counter Blast*:

"Have you not reason then to be ashamed," said he, addressing his subjects, "and to forbear this filthy novelty, so basely grounded, so foolishly received, and so grossly mistaken in the right use thereof. In your abuse thereof sinning against God, harming yourselves both in persons and goods, and taking also thereby the marks and notes of vanity upon you; by the custom thereof making yourselves to be wondered at by all foreign civil nations, and by all strangers, that come among you, to be scorned and contemned:* a custome loathsome to the eye, hatefull to the nose, harmeful to the brain, dangerous to the lungs, and in the black stinking fume thereof, nearest resembling the horrible Stygian pit that is bottomless."

King James mingled with his argument and his declamation, a plentiful seasoning of abuse. By him, and other writers of this period, who attacked Tobacco, it was constantly likened to the infernal regions. The smell of its smoke was even diabolical, and its effects were compared to the influence of the evil spirit.† Among others who wrote

---

* From hence, it may be inferred, that tobacco was, at this time, more used by the English than by other nations. Camden (*Life of Elizabeth*) says, that soon after the introduction of tobacco by the companions of Ralph Lane, it began to grow into great request, and to be sold at an high rate, whilst, in a short time, many men every where, some for wantonness, some for health sake, with insatiable desire and greediness, sucked in the stinking smoke thereof through an earthen pipe, which they presently blew out again at their nostrils; insomuch, that tobacco-shops are now as ordinary in most towns, as tap-houses and taverns. So that Englishmen's bodies, which are so delighted with this plant, seem, as it were, to be degenerated into the nature of barbarians.

"Tobacco, in our age," says Lord Bacon," is immoderately grown into use, and it affects men with a secret kind of delight, insomuch, that they who have once inured themselves to it, can hardly, afterwards, leave it; and, no doubt, it hath power to lighten the body and to shake off uneasiness."

† Sylvester compares the invention of gunpowder with smoking tobacco, of guns with tobacco-pipes! and adds, they were "*vented* from the infernal pit:" but thinks worse of tobacco than of gunpowder, because its operation is more insidious. Tobacconists he denominates idolaters; and, to end in an abusive climax on the smoke of tobacco, he introduces the following curious doggrell catalogue:—

There from a conviction of their own mind, or to flatter the prejudices of the monarch, *Joshua Sylvester* is most conspicuous. By his brethren of Parnassus this poet was called the silver-tongued Sylvester; and, perhaps, some passages in his translation of *du Bartas* entitle him to an epithet which could not have been allowed him, had his claim been founded only on the quibbling doggrell of the *Tobacco Battered.* Queen Elizabeth, it is said, had a great respect for the silver-tongued poet; King James had a greater; and Prince Henry greatest of all: who valued him so much, that he made him his first poet-pensioner. It ought not to be concealed, "that he was renowned by his virtuous fame; and by those of his profession, and such as admired poetry, esteemed a saint on earth, a true Nathaniel, a christian Israelite." But having been too boldly severe on the vices of the times, he was obliged to leave his native soil, and died at Middleburg, in Zealand, in 1618, at the age of fifty-five*. The *Tobacco-Battered,* allowing for poetical exaggeration, shows the extent of the influence this plant had attained; and how devoted the majority of mankind was to its soothing properties.† Ben Johnson, who was the friend of Sylvester,

---

There is first the *smoak* of Ignorance,
The *smoak* of Error, *smoak* of Arrogance,
The *smoak* of Merit super-er'gatory,
The *smoak* of Pardon, *smoak* of PURGATORY,
The *smoak* of Censing, *smoak* of *Thurifying*
Of Images, of *Satans Fury-flying,*
The *smoak* of Stewes (for *smoaking* thence they com,
As horrid hot as torrid *Sodom,* som):
Then *smoak* of *Powder-Treason, Pistols, Knives,*
To blow up Kingdoms, and blow-out Kings lives;
And lastly too TOBACCO's *smoaking-mists,*
Which (coming from *Iberian* BAALISTS)
No small addition of Adustion fit,
Bring to the smoak of the *unbottom'd* pit.

* Anthony Wood *(Athen. Oxon.)* the quaint biographer of this quaint poet, speaks of his head and heart in the highest terms of commendation. "He was very pious and sober; religious in himself and family, and courageous to withstand adversity: also he was adorned with the gift of tongues, French, Spanish, Dutch, Italian, and Latin." But he had not learned John Gay's prudential maxim,

"When you censure the age,
"Be cautious and sage,
"Lest the Courtiers offended should be."

† "O Great TOBACCO! greater than Great Can,
Great Turk, Great Tartar, or Great Tamerlane!
With Vultur's wings thou hast (and swifter yet
Than an Hungarian Ague, English Sweat)

Throughhurled his classic thunder against Tobacco; and never let pass an opportunity to abuse it. On all the modes of taking it, whether smoking, snuffing, or chewing, the poets have employed such punishment as the muse could inflict*. Neither have moral writers, nor even physicians been sparing of their censures.† But all objections have gone only to its indiscriminate use, or rather its abuse; and in no degree affect its character as a medicinal substance. A writer of the period when Tobacco was at the acmé of its influence, has brought together, into one paragraph, all its virtues and its vices, in a manner peculiar to himself.

"*Tobacco*, divine, rare, superexcellent Tobacco, which goes far beyond all their panaceas, potable gold, and philosophers stones, a sovereign remedy to all diseases. A good vomit, I confesse, a virtuous herb, if it be well qualified, opportunely taken, and medicinally used; but, as it is commonly abused by most men, which take it as tinkers do ale, it is a plague, a mischief, a violent purger of goods, lands, health; hellish, devilish and damned Tobacco, the ruine and overthrowe of body and soul."

With this curious summary of honest Burtons, I must conclude these miscellaneous Remarks on a Plant, whose dominion over mankind has been, for a period, most extensive; and trust to your indulgence, for the insertion, in your next number, of some observations on its *Botanical and Medical History*.

MEDICUS.

*November* 15, 1810.

---

Through all degrees flown far, nigh, up and down;
From Court to Cart; from Count to Country Clown,
Not scorning Scullions, Coblers, Colliers,
Jakes-farmers, Fidlers, Ostlers, Oysterers,
Roagues, Gypsies, Players, Panders, Punks; and all
What common Scums in common Sewers fall."
For all, as *Vassals*, at thy neck are bent,
And breathe by Thee, as their new *Element!*

* The Apostolic muse of Samuel Wesley thus attacks snuff-taking:
" To such a height with some is fashion grown,
They feed their very nostrils with a spoon.
One, and but one degree is wanting yet,
To make our senseless luxury complete;
Some choice regale useless as *snuff*, and dear,
To feed the mazy windings of the ear."

† Silvius de la Boe, and Kirkringius, were hostile to this plant; but the particulars of their hostility belong to its medical history.

